# Rare presentation of ileal mucosa-associated lymphoid tissue lymphoma: a case of obstructive syndrome in a Moroccan patient

**DOI:** 10.1093/jscr/rjaf534

**Published:** 2025-07-14

**Authors:** Imane Boujguenna, Mohamed Aziz Fadili, Hicham Krimou, Houcine Malki, Yassine Fakhri, Hind Boujguenna, Fatima Boukis, Samia Malki, Soufiane Abdouh, Nouhaila Haoussani, Ahmed Elguazzar

**Affiliations:** Guelmim Faculty of Medicine and Pharmacy, Ibn Zohr Agadir University Guelmim, Guelmim 81000 Morocco; General Surgery Department, Guelmim Military Hospital Moulay El Hassan, Guelmim 81000 Morocco; General Surgery Department, Guelmim Military Hospital Moulay El Hassan, Guelmim 81000 Morocco; Biology Department, Guelmim Military Hospital Moulay El Hassan, Guelmim 81000, Morocco; General Surgery Department, Guelmin Regional Hospital, Guelmim 81000, Morocco; Private University of Marrakesh, Marrakesh 40000, Morocco; Al AMAL Pathological Anatomy Laboratory, Guelmim 81000, Morocco; Mohammed VI Polytechnic University, Benguerir 43150, Morocco; Private Practice Physician, Marrakesh 40000, Morocco; Faculty of Medicine and Pharmacy of Rabat, University Mohammed V-Souissi, Rabat 10120, Morocco; Moulay Elhassan Military Hospital, General Surgery Department, Guelmim 81000, Morocco

**Keywords:** MALT, ileum, obstructive syndrome

## Abstract

The majority of extranodal lymphomas of the gastrointestinal tract involve the stomach and are often associated with *Helicobacter pylori* (*H. pylori*) infection. When lymphomas occur in the small intestine, diffuse large B-cell lymphomas are most common; however, mucosa-associated lymphoid tissue (MALT) lymphomas account for approximately one-third of small intestinal lymphomas. We report the case of an ileal MALT lymphoma in a 58-year-old Moroccan patient, characterized by its rare ileal localization and unusual presentation with an obstructive syndrome. Ileal MALT lymphoma is relatively uncommon compared to other gastrointestinal MALT lymphomas, such as gastric MALT lymphoma. Differentiating ileal MALT lymphoma from other tumors at this site based solely on clinical and radiological assessments can be challenging. Histopathological examination is pivotal for definitive diagnosis and for identifying underlying disease. Effective management relies on close collaboration among gastroenterologists, radiologists, surgeons, pathologists, and hematologists.

## Introduction

The majority of extranodal lymphomas of the gastrointestinal tract involve the stomach and are often associated with *Helicobacter pylori* (*H. pylori*) infection [1]. When lymphomas occur in the small intestine, diffuse large B-cell lymphomas are most common; however, mucosa-associated lymphoid tissue (MALT) lymphomas account for approximately one-third of small intestinal lymphomas [2]. We report the case of an ileal MALT lymphoma in a 58-year-old Moroccan patient, characterized by its rare ileal localization and unusual presentation with an obstructive syndrome.

## Case report

We describe a 58-year-old male patient, a chronic smoker with a history of treated pulmonary tuberculosis, who reported persistent, diffuse abdominal pain. This led to multiple consultations with general practitioners and specialists, as well as endoscopic examinations. Histopathological findings from these examinations suggested chronic inflammatory changes in the ileal and colonic mucosa with no signs of specificity or malignancy. The patient was managed symptomatically.

**Figure 1:**
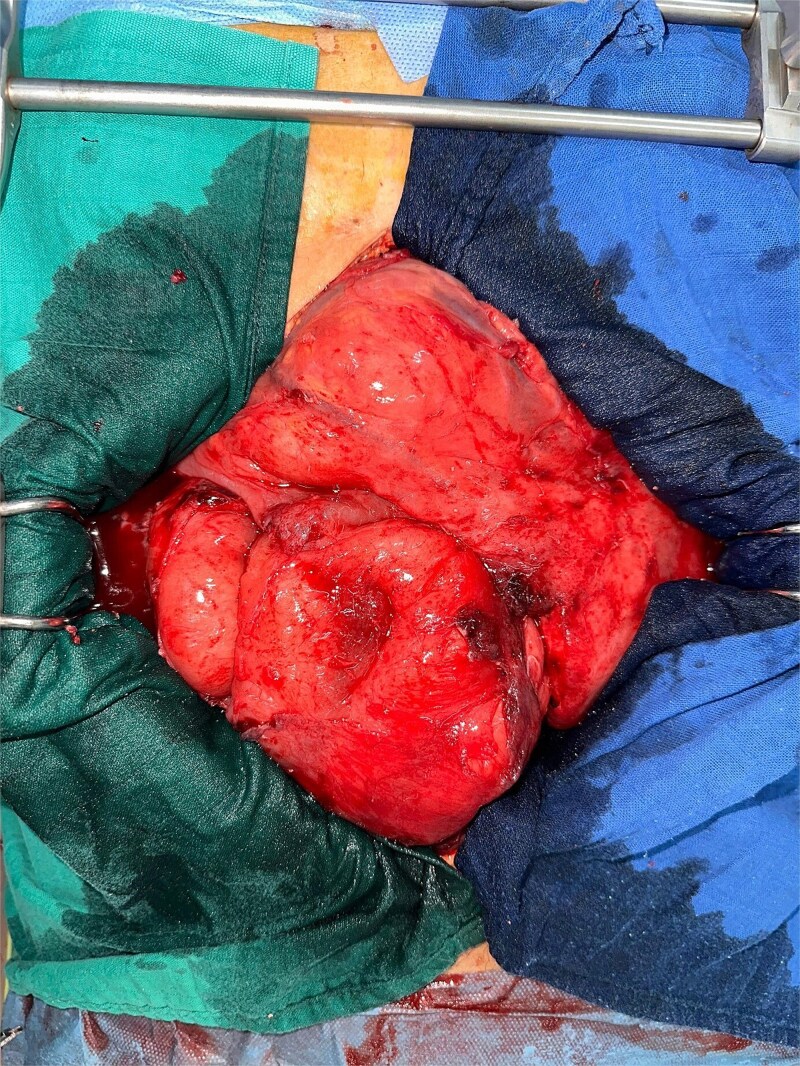
An omentum adherent to the underlying intestinal mass, and a conglomerate of agglutinated, obstructed small bowel loops with thickened, congested mesentery.

**Figure 2:**
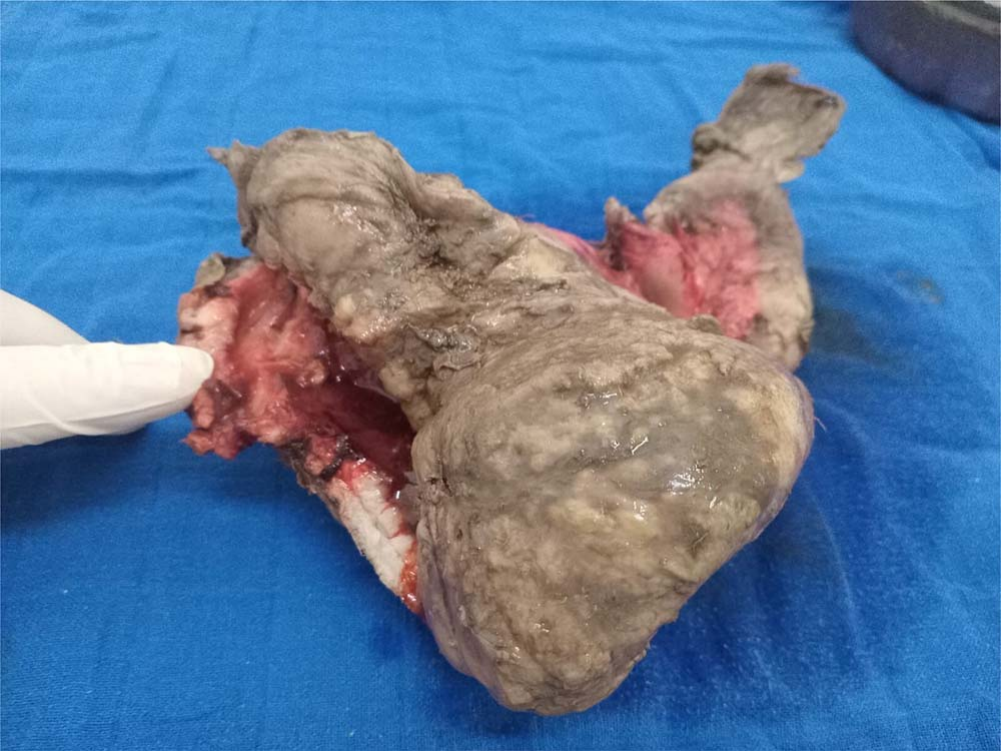
Intestinal mass.

**Figure 3:**
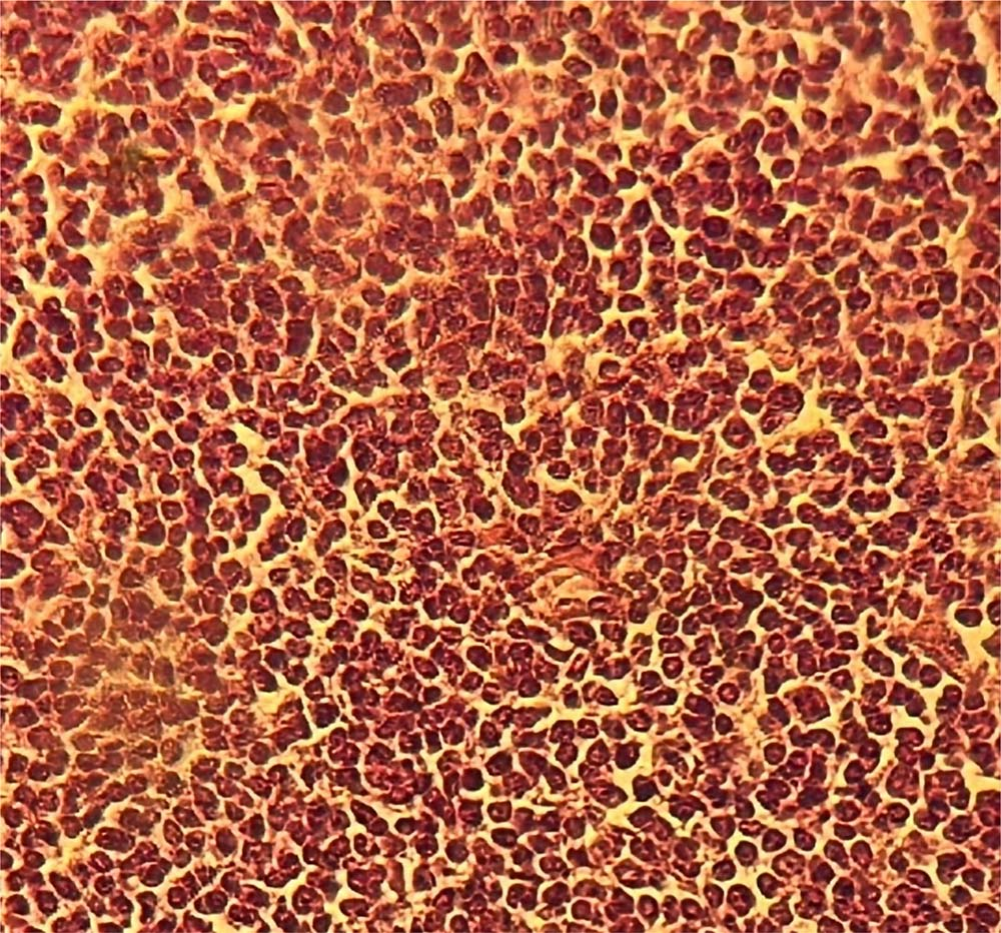
Dense lymphocytic infiltration.

**Figure 4:**
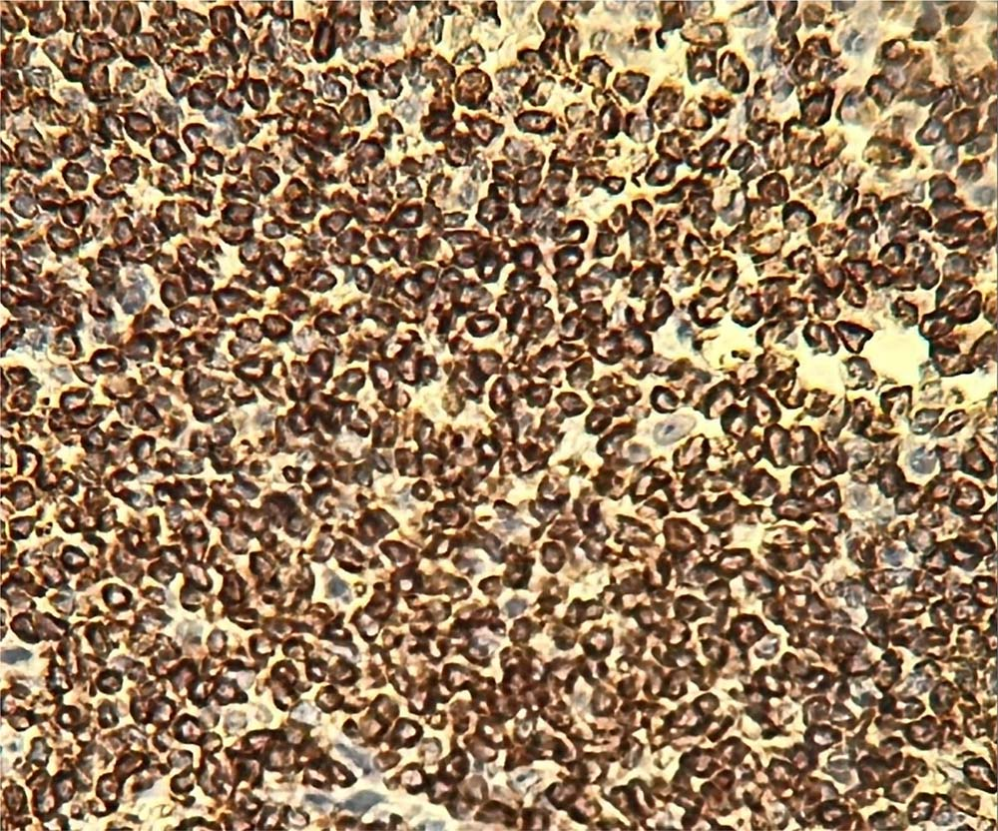
CD20.

In 2025, the patient presented with symptoms of bowel obstruction. Laboratory tests were unremarkable, but an abdominopelvic CT scan revealed small bowel obstruction associated with a tissue mass and significant ascites. Surgical exploration identified loculated serosanguinous ascites, an omentum adherent to the underlying intestinal mass, and a conglomerate of agglutinated, obstructed small bowel loops with thickened, congested mesentery (Fig. 1). The procedure included ascitic fluid aspiration, meticulous release of mid-small bowel loops, and resection of the conglomerate (Fig. 2) with the creation of a stoma. This presentation raised a differential diagnosis that included tuberculosis, Crohn’s disease, and lymphoma. Histopathological analysis revealed densely adhered small bowel loops without an identifiable mass. Microscopically, the intestinal wall exhibited dense lymphocytic infiltration (Fig. 3). Immunohistochemical studies showed positivity for CD20 (Fig. 4) and BCL2, with negativity for CK, CD5, Cyclin D1, CD10, CD138, and CD23, and a Ki67 index of 10%. Cytology indicated a reactive paucicellular pattern. Postoperative recovery was uneventful. The patient was referred to a hematologist, and a PET-CT scan revealed a hypermetabolic abdominal-pelvic focus with a SUVmax of 7.9, measuring 67 × 36 mm, along with splenic and gastric hypermetabolism. A multidisciplinary team recommended Rituximab (375 mg/m^2^ weekly) combined with *H. pylori* eradication. Follow-up imaging with CT was planned to evaluate therapeutic response.

## Discussion

In industrialized countries, small bowel obstruction is most commonly attributed to adhesions, accounting for ~70% of cases. Diffuse large B-cell lymphoma is the most frequent primary gastrointestinal lymphoma, with the small intestine as the site of origin in 15%–30% of cases [3]. MALT lymphoma of the small intestine remains rare [4].

Primary gastrointestinal lymphoma must meet the criteria defined by Dawson: (i) absence of peripheral lymphadenopathy at presentation, (ii) no mediastinal lymph node enlargement, (iii) normal total and differential leukocyte counts, (iv) predominant intestinal involvement at laparotomy with only adjacent lymph nodes affected, and (v) no hepatic or splenic lymphoma involvement.

Studies suggest that small intestinal MALT lymphomas are linked to chronic inflammatory bowel disease or malabsorption syndromes, such as *H. pylori*-associated gastritis or *Campylobacter jejuni* infection in the proximal small intestine. In our case, recurrent abdominal pain was reported with no identifiable underlying cause, and thorough macroscopic and microscopic examination of the surgical specimen did not reveal a specific etiology [5]. MALT lymphoma management primarily involves *H. pylori* eradication in the presence of bacterial involvement. In cases of refractory disease or absence of *H. pylori* infection, radiotherapy, chemotherapy, or anti-CD20 monoclonal antibody immunotherapy is considered. Radiotherapy shows excellent outcomes in localized cases, whereas disseminated or advanced disease warrants systemic approaches. Treatment should be individualized based on disease stage, symptoms, and patient preferences [6].

Ileal MALT lymphoma is exceptionally rare compared to other gastrointestinal MALT lymphomas, such as gastric MALT [7, 8]. Differentiating it from other ileal tumors based on clinical and radiological features alone poses challenges [9, 10]. Histopathological assessment remains crucial for diagnosis and for ruling out underlying disease. Optimal management requires coordinated efforts among gastroenterologists, radiologists, surgeons, pathologists, and hematologists.
